# The SR protein B52/SRp55 regulates splicing of the *period* thermosensitive intron and mid-day siesta in *Drosophila*

**DOI:** 10.1038/s41598-017-18167-3

**Published:** 2018-01-30

**Authors:** Zhichao Zhang, Weihuan Cao, Isaac Edery

**Affiliations:** 1 Rutgers University, Center for Advanced Biotechnology and Medicine, Piscataway, NJ 08854 USA; 2 Department of Molecular Biology and Biochemistry, Rutgers University, Center for Advanced Biotechnology and Medicine, Piscataway, NJ 08854 USA; 3 Present Address: Institute of Animal Genetics and Breeding, Sichuan Agriculture University, Huimin Road 211#, Chengdu, Sichuan Province 611130 P. R. China; 4 Present Address: Human Genetics Institute of New Jersey, Nelson Biology Laboratories, 604 Allison Road, Piscataway, NJ 08854 USA

## Abstract

Similar to many diurnal animals, *Drosophila melanogaster* exhibits a mid-day siesta that is more robust as temperature increases, an adaptive response that aims to minimize the deleterious effects from exposure to heat. This temperature-dependent plasticity in mid-day sleep levels is partly based on the thermal sensitive splicing of an intron in the 3′ untranslated region (UTR) of the circadian clock gene termed *period* (*per*). In this study, we evaluated a possible role for the serine/arginine-rich (SR) splicing factors in the regulation of dmpi8 splicing efficiency and mid-day siesta. Using a *Drosophila* cell culture assay we show that B52/SRp55 increases dmpi8 splicing efficiency, whereas other SR proteins have little to no effect. The magnitude of the stimulatory effect of B52 on dmpi8 splicing efficiency is modulated by natural variation in single nucleotide polymorphisms (SNPs) in the *per* 3′ UTR that correlate with B52 binding levels. Down-regulating *B52* expression in clock neurons increases mid-day siesta and reduces dmpi8 splicing efficiency. Our results establish a novel role for SR proteins in sleep and suggest that polymorphisms in the *per* 3′ UTR contribute to natural variation in sleep behavior by modulating the binding efficiencies of SR proteins.

## Introduction

Daily wake-sleep cycles in animals are governed by networks of cell-based circadian (≅24 hr) ‘clocks’ or pacemakers located in the brain^1,2^. Similar to many diurnal animals, the daily distribution of activity in *Drosophila melanogaster* exhibits a bimodal pattern with clock-controlled morning and evening peaks separated by a mid-day ‘siesta’^3^. Increases in daily temperature are accompanied by a gradual delay in the onset of the evening bout of activity and a more robust mid-day siesta^4–6^. Suppressing mid-day activity with a concomitant shift towards the cooler dusk hours on warm days minimizes the risks associated with exposure to the hot mid-day sun. We showed that this temperature-dependent behavioral adaptation is partially controlled by thermosensitive splicing of a 3′-terminal intron from the *Drosophila melanogaster period* (*dper*) transcript^5,7^, which is a key circadian clock factor known for encoding species-specific circadian behavioral programs in *Drosophila*^7–10^. The removal of this intron in the 3′ untranslated region (UTR) of *dper* RNA, named dmpi8 (*D*. *melanogaster per* intron 8), is inefficient at warmer temperatures, which attenuates the daily accumulation of *dper* mRNA, somehow leading to delayed evening activity and a more robust mid-day siesta^5,7^. The effect of dmpi8 splicing on mid-day activity levels involves a non-circadian mechanism that adjusts the daytime balance between sleep and wake-promoting pathways^11^. For example, on warm days the inefficient splicing of dmpi8 leads to an increase in arousal thresholds to light and other sensory-mediated cues, favoring sleep during the mid-day.

Splicing efficiency is dependent on the strengths of multiple *cis*-acting elements on precursor mRNA (pre-mRNAs) [i.e., 5′ splice site (ss), 3′ ss and branchpoint (BPS)], which influences the probability that they will be recognized by the spliceosome^12^. Temperature dependent splicing of dmpi8 was shown to result from suboptimal 5′ and 3′ splicing signals (ss), suggestion that splice site recognition/binding by the spliceosome to dmpi8 becomes progressively impaired as temperature increases^7^. Transgenic flies whereby the dmpi8 5′ and 3′ss were optimized exhibit near total removal of the dmpi8 intron at all temperatures and display less robust mid-day siestas compared to their wildtype control transgenics^7^.

**Figure 1:**
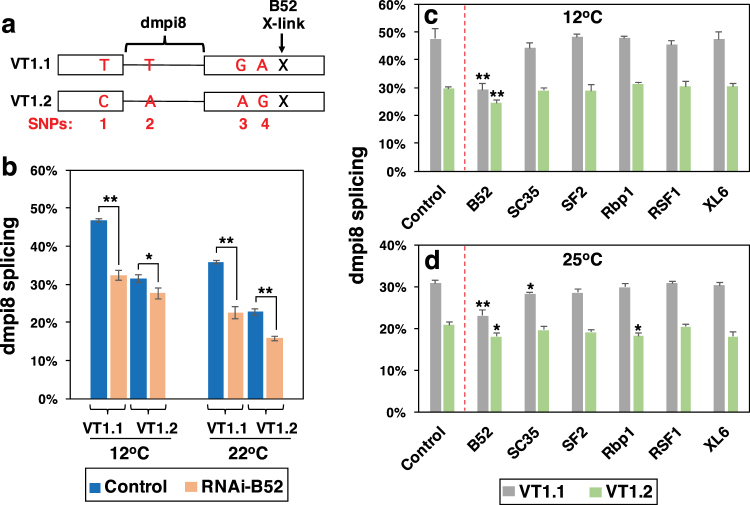
B52 stimulates dmpi8 splicing in *Drosophila* cultured cells. **(a)** Schematic diagram of the VT1.1 and VT1.2 haplotypes for the *dper* 3′ UTR showing the different SNP variants (red) and a previously identified B52 cross-linking site (X; Bradley *et al*.)^19^. **(b**–**d)** *Drosophila* S2 cells were transfected with either the pAct-Luc-VT1.1 (VT1.1) or pAct-Luc-VT1.2 (VT1.2) plasmid and grown at the indicated temperatures (12°, 22° or 25 °C). Cells were either mock-treated (control) or treated with double stranded RNA-mediated RNAi directed against the shown SR protein. RNA was purified from cell extracts and dmpi8 splicing efficiency calculated. Each experiment was done at least three times and values averaged. Values for dmpi8 splicing efficiency (% spliced) were significantly different between mock-treated (control) and RNAi-treated cells; *p < 0.05; **p < 0.01; two-tailed *t-*test. The following *p* values were determined (two-tailed *t*-test): [Panel (b); (1) 12 °C; VT1.1 control vs VT1.2 control, 4.2 × 10^−6^; VT1.1 control vs VT1.1 RNAi-*B52*, 3.6 × 10^−4^; VT1.2 control vs VT1.2 RNAi-*B52*, 0.045; (2) 22 °C; VT1.1 control vs VT1.2 control, 7.1 × 10^−4^; VT1.1 control vs VT1.1 RNAi-*B52*, 1.2 × 10^−3^; VT1.2 control vs VT1.2 RNAi-B52, 0.010]. [Panel (c); for VT1.1 control vs RNAi, B52, 0.012; SC35, 0.44; SF2, 0.89; Rbp1, 0.95; RSF1, 0.61; XL6, 0.97; for VT1.2 control vs RNAi, B52, 0.012; SC35, 0.65; SF2, 0.78; Rbp1, 0.10; RSF1, 0.63; XL6, 0.59]. [Panel (d); for VT1.1 control vs RNAi, B52, 0.006; SC35, 0.026; SF2, 0.10; Rbp1, 0.44; RSF1, 0.98; XL6, 0.58; for VT1.2 control vs RNAi, B52, 0.049; SC35, 0.33; SF2, 0.094; Rbp1, 0.040; RSF1, 0.62; XL6, 0.072].

Although weak 5′ and 3′ ss are the basis for the thermal sensitivity underlying dmpi8 splicing efficiency, analysis of natural populations of *D. melanogaster* derived from different continents identified several single nucleotide polymorphisms (SNPs) in the *dper* 3′ UTR that can modulate dmpi8 splicing efficiency and mid-day sleep^13,14^. This was first shown using independent isofemale lines established from natural populations of *D. melanogaster* that were originally caught along the eastern coast of the United States, extending from Florida to Vermont^14^. Sequencing of the *dper* 3′ UTR from various isofemale lines along this latitudinal cline identified four major SNPS (termed, SNPs 1–4) that generated two main *dper* 3′ UTR haplotypes, which we termed VT1.1 and VT1.2^14^ (see Fig. 1a). Natural populations of flies carrying the VT1.1 haplotype showed higher dimpi8 splicing efficiency and lower mid-day siesta compared to their VT1.2 counterparts. The enhanced splicing of dmpi8 in the VT1.1 context was recapitulated using a simplified *Drosophila* cell culture assay^14^. Transgenic flies whereby the only functional copy of *dper* carried the VT1.1 version of the *dper* 3′ UTR manifest higher dmpi8 splicing efficiency and reduced mid-day siesta compared to those with the VT1.2 haplotype^14^. Nonetheless, splicing of dmpi8 remains thermal sensitive for both VT1.1 and VT1.2 because they have the identical 5′ and 3′ ss^14^. Thus, while the weak 5′ and 3′ ss ensure that dmpi8 splicing efficiency is thermal sensitive, wild-derived SNPs in the *dper* 3′ UTR can alter the baseline splicing efficiency of dmpi8, resulting in natural variation in mid-day siesta levels.

Besides *cis*-acting elements such as the strengths of key splicing signals and SNPs, *trans*-acting factors beyond the core spliceosome might also regulate dmpi8 splicing efficiency. An important class of splicing factors are the highly-conserved serine/arginine (SR) family of proteins^15–17^. SR proteins have RNA-recognition motifs (RRMs) that bind specific *cis*-acting elements on precursor mRNAs (pre-mRNAs) and can enhance or repress splicing events via RS-motif mediated protein-protein interactions^18^. In this study, we sought to determine if one or more SR proteins modulate mid-day siesta and/or the splicing efficiency of the dmpi8 intron. There are eight identified SR proteins in *Drosophila* that also have close homologs in mammals (SC35, SF2, SRp54, XL6, Rbp1, B52, Rsf1, and Rbp1-like)^19,20^. Using a simplified *Drosophila* cell culture system^7,14^, we show that B52/SRp55 enhances the splicing efficiency of dmpi8, with larger effects in the VT1.1 context compared to that of VT1.2. In agreement with these results, cross-linking and immunoprecipitation studies revealed that although B52 binds transcripts containing either the VT1.1 or VT1.2 versions of the *dper* 3′ UTR, binding levels are higher for transcripts containing the VT1.1 variant. Consistent with a stimulatory role for B52 in dmpi8 splicing, down-regulating *B52* expression in clock neurons increases mid-day siesta with little effect on nighttime sleep levels. Our findings identify B52 as a novel regulator of sleep/arousal in *Drosophila*, and suggest that the interplay between *cis*-acting SNPs and *trans*-acting SR proteins contributes to natural variation in sleep behavior.

## Results

### Down-regulating *B52* in *Drosophila* cultured cells reduces dmpi8 splicing efficiency, with larger magnitude effects observed with the VT1.1 haplotype

In prior work, we developed a simplified *Drosophila* cell culture system that can recapitulate the thermal sensitive splicing of the dmpi8 intron^7^. In this assay, *Drosophila* Schneider 2 (S2) cells are transiently transfected with plasmids that contain the *luciferase* (*Luc*) open reading frame followed by the entire d*per* 3′ UTR, including ~90 bp of proximal 3′ non-transcribed sequences. Expression of *Luc-dper* 3′ UTR transcripts is driven by the constitutive pAct promoter. Cells are grown at different temperatures, extracts prepared and dmpi8 splicing efficiency determined using RT-PCR. We tested the effects of SR proteins on the splicing efficiency of dmpi8 within the VT1.1 and VT1.2 *dper* 3′ UTR haplotypes (Fig. 1a). In agreement with prior work, the splicing efficiency of dmpi8 in the Luc-VT1.1 construct is approximately 35% higher compared to the Luc-VT1.2 version (Fig. 1b; compare control levels; two-tailed *t*-test, *p* value = 4.2 × 10^−6^ for 12 °C; *p* value = 7.1 × 10^−4^ for 22 °C)^14^. Also, as previously noted, dmpi8 splicing for both haplotypes is enhanced at cold temperatures (Fig. 1b–d)^14^. Thermal sensitive splicing of dmpi8 is based on the suboptimal 5′ and 3′ ss, which are identical for dmpi8 in both VT1.1 and VT1.2^7,14^. Therefore, although the VT1.1 and VT1.2 backgrounds differentially affect baseline levels of dmpi8 splicing, removal of this intron remains relatively inefficient and thermo-sensitive for both haplotypes.

To identify SR proteins that might regulate dmpi8 splicing efficiency, we reduced their levels using RNAi-mediated knockdown and compared the splicing efficiency of dmpi8 in the Luc-VT1.1 and Luc-VT1.2 contexts. In all, we analysed a total of six different *Drosophila* SR proteins (B52, SC35, SF2, Rbp1, RSF1 and XL6). Prior work showed that all eight SR proteins are endogenously expressed in *Drosophila* S2 cells^19,21,22^. Of the SR proteins tested, only reducing *B52* (*Drosophila* homolog of SRp55) consistently led to significant changes in dmpi8 splicing efficiency (Fig. 1b–d; see figure legend for *p* values). Knock-down of *B52* expression led to an approximately 25% reduction in the splicing efficiency of dmpi8 in the VT1.1 background, a response observed over a wide range of temperatures (Fig. 1b–d; and data not shown). With regards to VT1.2, *RNAi-B52* treatment also inhibits dmpi8 splicing, but in a manner that is generally more modest compared to VT1.1 (Fig. 1b–d). Together, our findings reveal a role for B52 in regulating dmpi8 splicing, and suggest that *dper* transcripts with the VT1.1 haplotype are better targets for B52 activity compared to those with the VT1.2 version (see below).

The demonstration that B52 can regulate dmpi8 splicing efficiency is consistent with a transcriptome wide analysis of SR protein binding to transcripts expressed in *Drosophila* S2 cells. Using cross-linking and immunoprecipitation coupled with high-throughput sequencing (iCLIP-seq), they identified a B52 binding site slightly downstream of the closely spaced SNPs 3 and 4 in the *dper* 3′ UTR (^19^; and T. Bradley and M. Blanchette, personal communication) (see Fig. 1a).

### Binding of B52 to transcripts containing the VT1.1 *dper* 3′ UTR is significantly higher compared to those with the VT1.2 haplotype

**Figure 2:**
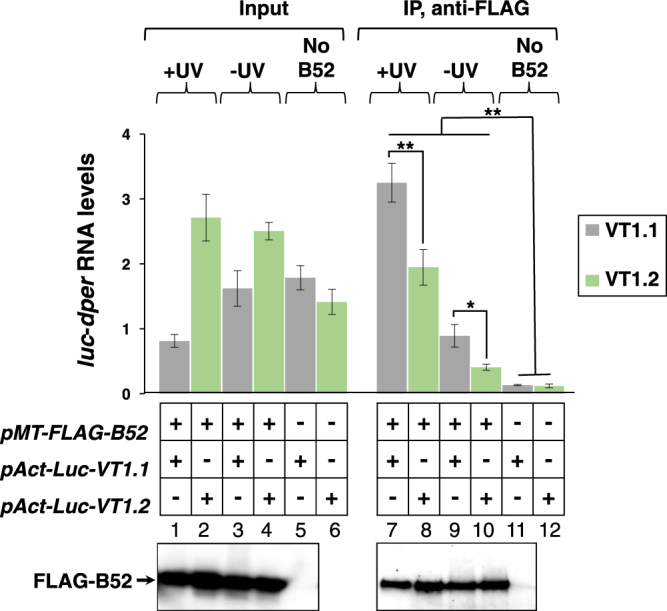
Enhanced binding of B52 to transcripts containing the VT1.1 haplotype compared to the VT1.2 version. *Drosophila* S2 cells were transfected with either the the pAct-Luc-VT1.1 (VT1.1) or pAct-Luc-VT1.2 (VT1.2) plasmid, in the presence (+) or absence (−) of a plasmid expressing FLAG-B52, as indicated. After UV irradiation (+UV) or mock-treatment (−UV), cells were homogenized in 350 μl of lysis buffer. A fraction of the cell extract (100 μl) was used to measure the relative levels of *Luc-dper* transcripts (Input; top left) and FLAG-B52 using immunoblotting (Input; bottom left, lanes 1–5). The remainder of the cell extract was subjected to immunoprecipitation (IP) in the presence of anti-FLAG antibodies. Following IP, an aliquot was used for immunoblotting of FLAG-B52 (IP; bottom right, lanes 7–11), and the remainder used to measure the relative levels of *Luc-dper* transcripts bound to B52 (IP; top right). For the immunoblots shown, the left panel (lanes 1–5) and the right panel (lanes 7–11) come from two different gels (for images of the full-length blots, see figure S1). Note that for the set of immunoblots shown we did not include extracts prepared from the control samples expressing pAct-Luc-VT1.2 in the absence of FLAG-B52 (lanes 6 and 12). For quantitation of transcript levels, results from three experiments were averaged. The values for *Luc-dper* mRNA bound to B52, either with or without cross-linking, was significantly higher for VT1.1 compared to VT1.2, even though the starting amount of VT1.2 RNA was generally higher compared to VT1.1 (Input); the following *p* values were determined for IP samples comparing VT1.1 and VT1.2 values: +UV, 0.0035; −UV, 0.043. Also, the levels of *Luc-dper* transcripts present in immune complexes for cells expressing FLAG-B52 were significantly higher compared to negative control samples derived from cells not expressing FLAG-B52. The following *p* values were obtained comparing either *Luc-VT1.1* or *Luc-VT1.2* transcript levels in immune complexes expressing FLAG-B52 (lanes 7–10) compared to control samples not expressing FLAG-B52 (lanes 11 and 12); VT1.1/+UV vs VT1.1/No B52, 0.003; VT1.1/−UV vs VT1.1/No B52, 0.01; VT1.2/+UV vs VT1.2/No B52, 0.005; VT1.2/−UV vs VT1.2/No B52, 0.0004. *p < 0.05; **p < 0.01; two-tailed *t-*test.

Encouraged by the prior finding of a B52 binding site in the *dper* 3′ UTR^19^, we sought to use the same experimental strategy and compare the relative binding levels of B52 to transcripts containing either the VT1.1 or VT1.2 versions of the *dper* 3′ UTR. To this end we transfected cells with an amino-terminal FLAG-tagged version of B52 (FLAG-B52) and co-transfected plasmids containing either the Luc-VT1.1 or Luc-VT1.2 plasmids. Cells were exposed to short-term treatment with UV irradiation to stabilize protein-RNA interactions by inducing covalent cross-links. Subsequently, FLAG-B52 was immunoprecipitated (IP) and the levels of the *Luc-dper* transcripts (Fig. 2, top) and FLAG-B52 (Fig. 2, bottom; and see Fig. S1) present in immune-complexes were measured.

B52 interacts with both VT1.1 and VT1.2-containing transcripts, however binding levels are approximately 40% higher with the VT1.1 version (*p* = 0.0035, two-tailed *t*-test; Fig. 2 top, compare lanes 7 and 8). The higher amounts of B52 bound to VT1.1 was not due to significant differences in FLAG-B52 levels as immunoblotting clearly showed that similar amounts of recombinant B52 were produced and immunoprecipitated for cells transfected with either Luc-VT1.1 or Luc-VT1.2 (Fig. 2, bottom panels, lanes 1–4 and 7–10; see also Fig. S1). Similar results were also obtained in the absence of UV irradiation (Fig. 2 top, compare lanes 9 and 10), although as might be expected the total levels of *Luc-dper* transcripts bound to B52 were reduced compared to cells treated with UV irradiation (compare lanes 9 and 10 to 7 and 8). Several control experiments showed that the presence of either *Luc-VT1.1 or Luc-VT1.2* transcripts in immune complexes was highly dependent on the presence of FLAG tagged B52 (Fig. 2, compare lanes 11 and 12 to lanes 7–10; and data not shown). The binding studies are very consistent with the RNAi results (Fig. 1), and strongly suggest that the higher levels of B52 bound to the VT1.1 haplotype contribute to its increased dmpi8 splicing efficiency compared to VT1.2 (see Discussion).

### Knock down of *B52* in clock cells leads to increased mid-day sleep in flies

To test the physiological significance of the results we obtained in cultured cells, we used the UAS/GAL4 binary system^23,24^ to knock down endogenous expression of SR proteins in a tissue-specific manner and measure the effects on daily wake-sleep cycles. Flies expressing RNAi against SR proteins were obtained from several public stock centers and crossed to flies bearing several tissue-specific *Gal4* drivers that express in the head. B52 is abundantly expressed in the brain and eyes (^25^; and annotated data from flybase.org), and the brain is the main anatomical location regulating daily wake-sleep behaviour^26^. In addition, we also set-up control crosses for each of the respective drivers and RNAi lines. All the flies used were in the standard *w*^1118^ genetic background. To enhance the effectiveness of RNAi-mediated inhibition we co-expressed a *UAS-dicer2* (*UAS-Dcr-2*) transgene^27^. For each RNAi line, we pooled behavioral data from two reciprocal crosses; i.e., in one set we crossed male flies carrying tissue-specific drivers with female virgins carrying RNAi lines, and in the other set we did the opposite cross. Young adult progeny from the crosses were entrained to standard conditions of 12 hr light: 12 hr dark [LD; where zeitgeber time (ZT) 0 is lights-on and ZT12 is lights-off] at either 18° or 25 °C for 4–5 days, followed by 5–7 days of total darkness (DD). Daily wake-sleep behaviour was recorded using the standard Trikinetics system^28,29^. In LD cycles, flies manifest clock-controlled morning and evening bouts of activity separated by a mid-day dip in activity levels or siesta (e.g., see Figs S2 and S4). Since down-regulating *B52* in cultured cells led to a decrease in dmpi8 splicing efficiency (Fig. 1), we predicted that the same treatment in flies would increase daytime sleep levels.

**Figure 3:**
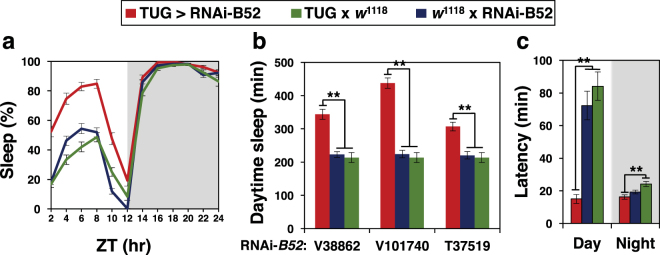
Knock down of *B52* in clock cells preferentially modulates daytime sleep during a daily light-dark cycle. **(a–c)** Young male progeny from the indicated cross (top of panels) were kept at 18 °C and entrained for 5 days in 12 hr:12 hr light/dark (LD) cycles [where Zeitgeber time (ZT) 0 is lights-on] followed by several days in constant darkness (DD; see Fig. 4). Daily wake sleep levels **(a)**, total daytime sleep **(b**) and sleep latency **(c)** were measured, and average values during the last 3 days of LD are shown. For each genotype, data from 16 individual flies was used. Daily sleep levels **(a)** and sleep latency **(c)** for *TUG > RNAi-B52(V101740)* and control crosses are shown. Similar results were obtained with other RNAi-B52 lines (data not shown). **(b)** Total daytime sleep levels (ZT0–12) for several independent lines of RNAi-B52 (V38862, V101740, T37519) and appropriate control crosses. The following *p* values were determined: [Panel (a), ANOVA, TUG > V101740 compared to both controls; for daytime values from ZT0-12, 3.4 × 10^−6^; for nighttime values from ZT12-24, 0.057]. [Panel (b), two-tailed *t*-test; TUG > V38862 vs V38862 x *w*^1118^, 5.0 × 10^−10^; TUG > V38862 vs TUG x *w*^1118^, 1.2 × 10^−8^; TUG > V101740 vs V101740 x *w*^1118^, 1.68 × 10^−20^; TUG > V101740 vs TUG x *w*^1118^, 1.96 × 10^−18^; TUG > T37519 vs T37519 x *w*^1118^, 2.19 × 10^−6^; TUG > T37519 vs TUG x *w*^1118^, 1.06 × 10^−8^]. [Panel (c), two-tailed *t*-test; for day values, TUG > V101740 vs V101740 x *w*^1118^, 9.56 × 10^−8^; TUG > V101740 vs TUG x *w*^1118^, 4.8 × 10^−10^; for night values, TUG > V101740 vs V101740 x *w*^1118^, 0.081; TUG > V101740 vs TUG x *w*^1118^, 0.00025]. (**b,c**) Values for TUG > RNAi-B52 were significantly different compared to one or more control crosses; *p < 0.05; **p < 0.01; two-tailed *t-*test. The corresponding daily activity profile for panel (a) is shown in Figure S2a.

Indeed, down-regulation of *B52* in clock cells using the standard pan-clock driver termed *tim-UAS-Gal4* (*TUG*)^30^ led to a significant increase in day-time sleep compared to the control crosses, with little to no effect on night-time sleep levels (Fig. 3, a and b; see figure legend for *p* values). Increased daytime sleep in flies expressing *RNAi-B52* in clock cells (*TUG* > *RNAi-B52*) with little effect on night-time sleep levels was observed for several independent *RNAi-B52* lines (Fig. 3b and data not shown). Although most of our behavioural studies were done with males, inhibiting *B52* also increased daytime sleep in females (Fig. S3). Knock-down of *B52* augmented daytime sleep levels at both 18° and 25 °C, although for males this was more readily observed at the cooler temperature (Fig. S3, compare panels a and b). This is not surprising because there is sexual dimorphism in mid-day sleep, whereby males sleep more compared to females^4,29^ (Fig. S3, compare panels a to c, and b to d). Thus, at higher temperatures where mid-day sleep levels are already high in males, this likely produces a ‘ceiling’ effect, minimizing the ability of silencing *B52* to further increase daytime sleep levels. Nonetheless, despite some differences in the overall magnitude of daytime sleep increases by knock-down of *B52*, the response is very robust, occurring with numerous independent RNAi lines, in both sexes and at multiple temperatures.

**Figure 4:**
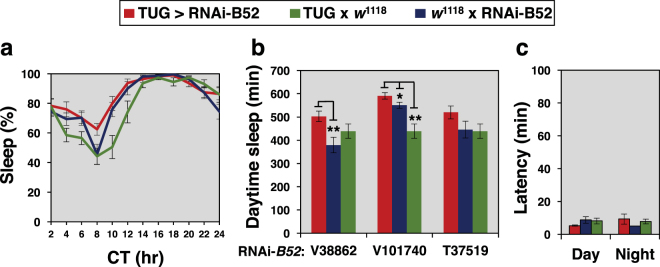
The effect of B52 on daily sleep levels is strongly reduced in constant dark conditions. **(a–c**) Young male progeny from the indicated cross (top of panels) were kept at 18 °C and entrained for 5 days in 12 hr:12 hr light/dark (LD) cycles followed by several days in constant darkness [where circadian time (CT) 0 is the beginning of the subjective day; i.e., herein defined as equivalent to ZT0 in LD]. Figures are derived from the same data shown in Fig. 3, except that data are from the first day of constant darkness based on averaging values obtained from 16 individual flies for each genotype. The following *p* values were determined: [Panel (a), ANOVA, TUG > V101740 compared to both controls, 0.13]. [Panel (b), two-tailed *t*-test; TUG > V38862 vs V38862 x *w*^1118^, 4.3 × 10^−3^; TUG > V38862 vs TUG x *w*^1118^, 0.11; TUG > V101740 vs V101740 x *w*^1118^, 0.022; TUG > V101740 vs TUG x *w*^1118^, 9.38 × 10^−5^; TUG > T37519 vs T37519 x *w*^1118^, 0.13; TUG > T37519 vs TUG x *w*^1118^, 0.079]. [Panel (c), two-tailed *t*-test; for day values, TUG > V101740 vs V101740 x *w*^1118^, 0.12; TUG > V101740 vs TUG x *w*^1118^, 0.16; for night values, TUG > V101740 vs V101740 x *w*^1118^, 0.15; TUG > V101740 vs TUG x *w*^1118^, 0.65]. The corresponding daily activity profile for panel (a) is shown in figure S2b.

Strong daily wake-sleep rhythms with ~24 hr periods were observed with RNAi-mediated knock-down of *B52* in clock cells (Table 1). In some cases we noted that expressing *RNAi-B52* in clock cells led to increases in period length compared to one or more control crosses (Table 1). Although this might suggest a role for B52 in circadian regulation, we observed robust effects on mid-day sleep that were not accompanied by period changes (Fig. 3 and Table 1), indicating the sleep effects are likely unrelated to circadian properties. Indeed, in complete darkness we observed smaller or no effects on daytime sleep levels by driving *RNAi-B52* expression in clock cells compared to control crosses (Fig. 4). A much reduced effect of knocking down *B52* on sleep levels during constant darkness is consistent with prior work showing that dmpi8 splicing mainly regulates daytime sleep behaviour by adjusting arousal thresholds to sensory mediated cues, such as photic signals^11^. Activity levels during wake periods showed little difference between *TUG* > *RNAi-B52* flies and control crosses (data not shown), indicating that changes in sleep behaviour are not due to gross health issues.

**Table 1:** Rhythmic behavior of flies expressing RNAi-B52 in *tim*-expressing cells and control crosses.

Genotype^a^	°C	n^b^	Rhythmic (%)	Period^c^ ± SEM	Power^d^ ± SEM
TUG x *w*^1118^	18	30	100	24.4 ± 0.2	222.2 ± 20.6
V38862 x *w*^1118^	18	28	93.8	24.2 ± 0.3	249.0 ± 19.8
V101740 x *w*^1118^	18	30	100	23.9 ± 0.1	268.3 ± 13.6
T37519 x *w*^1118^	18	27	100	23.8 ± 0.2	198.9 ± 14.7
TUG x V38862	18	23	85.4	23.9 ± 0.2	251.1 ± 24.8
TUG x V101740	18	24	91.2	25.6 ± 0.3	188.2 ± 15.2
TUG x T37519	18	19	91.7	24.6 ± 0.6	165.6 ± 21.4
TUG x *w*^1118^	25	31	100	24.0 ± 0.1	286.8 ± 12.5
V38862 x *w*^1118^	25	30	100	23.7 ± 0.04	314.5 ± 14.8
V101740 x *w*^1118^	25	32	100	23.8 ± 0.06	309.1 ± 15.3
T37519 x *w*^1118^	25	29	100	23.8 ± 0.09	256.8 ± 17.7
TUG x V38862	25	27	100	23.5 ± 0.07	293.5 ± 19.5
TUG x V101740	25	32	100	24.8 ± 0.2	268.7 ± 20.5
TUG x T37519	25	30	93.3	24.2 ± 0.1	219.0 ± 19.4

^a^For each cross we set up two contemporaneous crosses using males from one genotype crossed to virgin females of the other genotype and vice-versa. All results are an average of crosses in both directions. Independent lines of *RNAi-B52* were used (V38862, V101740, T37519). All flies were in the *w*^1118^ genetic background and the TUG flies co-expressed *UAS-Dcr-2*.

^b^Number of flies that survived the entire testing period.

^c^Flies were entrained for 5 days in LD at the indicted temperature followed by 7 days in complete darkness (DD). Activity data collected during DD was used to calculate free-running periods.

^d^Power is a relative measure of rhythm strength.

In addition, we also measured the timing of the first sleep bout following lights-on (day-latency) and lights-off (night-latency). Flies expressing *RNAi-B52* in clock cells began sleeping much earlier following lights-on compared to appropriate controls (i.e., begin mid-day siesta earlier), whereas the timing of the first night-time sleep (night-latency) showed little to no difference from the control crosses (Fig. 3c). As with daytime sleep levels, the effect of knocking down *B52* on sleep latency (Fig. 3c) was much reduced or not observed in constant darkness (Fig. 4c). This further establishes a preferential role for B52 in regulating daytime sleep behaviour.

**Figure 5:**
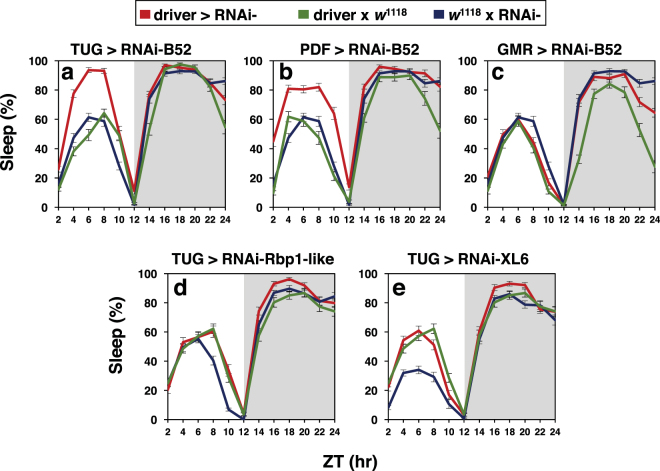
Modulation of daily sleep levels is not a shared feature of all SR proteins. **(a–e**) Young male progeny from the indicated crosses (top of panels) were kept at 18 °C and entrained for 5 days in 12 hr:12 hr light/dark (LD) cycles followed by several days in constant darkness (DD). Shown are the daily sleep levels (averaged over the last 3 days of LD). For each genotype, data from 16 individual flies was used to generate the graphs shown in each panel. The driver and SR protein targeted by RNAi are indicated. For RNAi-B52, the V101740 line is shown. The following *p* values (ANOVA) were determined for driver x RNAi (red) compared to both control crosses (blue and green): [daytime values from ZT0-12; panel (a), 0.0040; panel (b), 0.00039; panel (c), 0.92; panel (d), 0.48; panel (e), 0.28; nighttime values from ZT12–24; panel (a), 0.36; panel (b), 0.015; panel (c), 0.26; panel (d), 0.021; panel (e), 0.13]. The corresponding daily activity profiles are shown in Figure S4.

There are approximately 150 *dper/tim*-expressing clock neurons in the adult fly brain that regulate circadian rhythms and sleep^26,31^. Amongst these, the pigment dispersing factor (PDF)-expressing cells comprise a small set of key brain pacemaker neurons that are not only central to maintaining free-running wake-sleep rhythms^32^ but also modulate arousal and sleep^33–36^. Day-time sleep levels were also significantly increased when knock down of *B52* was limited to the PDF-expressing clock cells (Fig. 5b; see figure legend for *p* values; see Fig. S4 for daily activity profiles), suggesting a prominent role for these cells in regulating mid-day siesta. In contrast, down regulating *B52* in photoreceptor cells (*Gmr-Gal4*) did not significantly alter daily sleep patterns compared to both control crosses (Fig. 5c). Furthermore, RNAi directed against several other SR proteins had no significant effects on daily sleep levels relative to both control crosses even when driven by the TUG driver (Fig. 5d,e; and data not shown). Thus, although we did not do an extensive analysis of tissue-specific drivers and SR proteins, the results establish a role for B52 in clock cells as a regulator of mid-day siesta levels, a role that does not appear to be a shared function of all SR proteins. Several attempts at overexpressing B52 using a variety of drivers rendered the flies sick, making it difficult to evaluate possible effects on sleep (data not shown).

### B52 stimulates dmpi8 splicing in flies

**Figure 6:**
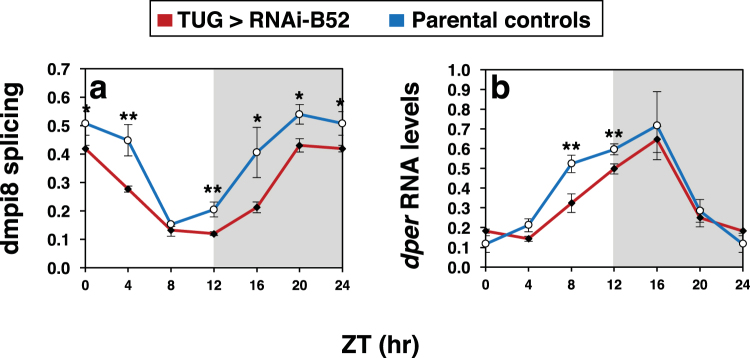
B52 stimulates dmpi8 splicing in flies. **(a,b**) Crosses were set up between the TUG driver and independent lines of *RNAi-B52* (V38862, V101740 and T37519). For each RNAi line, we set up two crosses; in one set we used male TUG and female virgin RNAi lines and in the other set we did a reciprocal cross. For each cross, young adult progeny were placed in 6 vials (each vial had ~40 flies), and entrained for 5 days in 12 hr:12 hr light/dark (LD) cycles at 25 °C. In addition, the same was done for the parental controls (TUG, V38862, V101740 and T37519). On the last day of LD, at the indicated times, 1 vial for each cross was collected by freezing. For each RNAi line, flies from both crosses were pooled. Total RNA was extracted from fly heads and the splicing efficiency of dmpi8 (**a**) and total levels of *dper* (**b**) were calculated. Because results were highly similar for the different *RNAi-B52* line and their parental controls (Fig. S5), we averaged all three TUG > RNAi-B52 crosses, and pooled the data from the parental controls to yield the group averages shown. Values shown for dmpi8 splicing efficiency (shown as fraction, where 1.0 is equal to 100% splicing of dmpi8) and *dper* RNA levels are from the average of two independent experiments. Note that the values for ZT0 were re-plotted for ZT24. (**a**) The daily dmpi8 splicing efficiency for flies expressing RNAi-B52 in *tim*-expressing cells (TUG > RNAi-B52) was significantly different compared to the parental controls (ANOVA, *p* = 0.010); in addition, for each time point we determined *p* values (two-tailed *t*-test); ZT0/24, 0.027; ZT4, 0.0054; ZT8, 0.45; ZT12, 0.0051; ZT16, 0.031; ZT20, 0.026. (**b**) For *dper* RNA levels we determined *p* values for each time point (two-tailed *t*-test); ZT0/24, 0.23; ZT4, 0.15; ZT8, 0.009; ZT12, 0.004; ZT16, 0.23; ZT20, 0.60. *p < 0.05; **p < 0.01; two-tailed *t-*test. The data clearly show that knock down of *B52* in *tim*-expressing cells reduces the daily splicing efficiency of dmpi8.

Based on the behavioural findings, we analysed the daily splicing efficiency of dmpi8 in head extracts prepared from flies expressing *RNAi-B52* in clock cells and the results were compared to contemporaneously treated parental controls. We performed several experiments and evaluated different independent *RNAi-B52* lines relative to parental controls. Because the different RNAi-B52 lines and parental controls yielded similar results (Fig. S5), we pooled the data (Fig. 6). As previously reported, the splicing efficiency of dmpi8 in fly heads undergoes daily rhythms during a daily light-dark cycle, reaching trough levels during the late day/early night (Fig. 6a)^37,38^. The results clearly show that the overall daily splicing efficiency of dmpi8 is reduced in flies expressing *RNAi-B52* in clock cells compared to parental controls (Fig. 6a and data not shown; ANOVA, *p* = 0.01). While knock-down of *B52* in clock cells reduced the overall daily splicing efficiency of dmpi8 it did not alter its cycling pattern, consistent with a clock-independent role for B52 in regulating daytime sleep levels. We also measured the total levels of *dper* mRNA, which undergo daily cycles in abundance^39^. Knock-down of *B52* led to modest reductions in the overall daily levels of *dper* transcripts, especially during its daily upswing (Fig. 6b). These results are consistent with prior work showing that decreases in dmpi8 splicing efficiency reduce *dper* mRNA levels via a mechanism that is not well understood^5^.

Finally, we sequenced the *dper* 3′ UTR in the different RNAi and driver lines used in our behavioral analysis. All carried the VT1.2 version of the *dper* 3′ UTR (data not shown). At present, it is not clear if larger effects on day-time sleep and/or dmpi8 splicing would be observed by knock-down of *B52* in a VT1.1 genetic background. Irrespective, results from both *Drosophila* cultured cells and in flies indicate that B52 is an activator of dmpi8 splicing (Figs 1 and 6), consistent with its preferential effects on daytime sleep (e.g., Figs 3–5 and S3).

## Discussion

SR proteins are members of a family of splicing regulatory factors that are highly conserved from *Drosophila* to humans^20,40^. In this report, we examined potential roles for SR proteins in regulating dmpi8 splicing and mid-day siesta in *D. melanogaster*. Studies in S2 cells and flies show that B52 stimulates dmpi8 splicing efficiency, and is a novel sleep/arousal factor regulating mid-day siesta levels. Down regulation of *B52* in clock cells increased mid-day sleep levels with little to no effect on night sleep (Fig. 3a,b, and S3). In addition, mid-day siesta began significantly earlier in flies with knock down of *B52* in clock cells compared to control crosses (Fig. 3c). The effects of inhibiting *B52* expression in clock cells on daytime sleep levels and sleep latency were greatly reduced or absent in complete darkness (Fig. 4).

Together, the sleep phenotypes resulting from down-regulating *B52* are remarkably similar to what we previously reported using transgenic flies where we modified the splicing efficiency of dmpi8^11^. For example, increasing the splicing efficiency of dmpi8 reduces mid-day sleep levels in daily light-dark cycles but not complete darkness^11^. Thus, even though knock-down of *B52* also lowers dmpi8 splicing efficiency during the night (Fig. 6a), the preferential effect of this treatment on daytime sleep levels (Figs 3 and 5a) is consistent with prior work showing that dmpi8 splicing does not alter baseline sleep levels but modulates arousal thresholds to light and other sensory-mediated cues^11^. Although SR proteins have other functions in RNA metabolism and gene expression besides splicing^16,40,41^, the remarkably parallel sleep effects observed with knock-down of *B52* in adult pacemaker cells compared to those obtained with altering dmpi8 splicing efficiency in transgenic models strongly suggest that B52 mainly modulates mid-day sleep levels via regulating dmpi8 splicing efficiency. Moreover, the splicing efficiency of dmpi8 is causally linked to changes in *dper* mRNA levels, whereby reducing dmpi8 splicing leads to decreases in the abundance of *dper* transcripts^5,7,38^. Likewise, silencing *B52* in clock cells not only decreases dmpi8 splicing efficiency (Fig. 6a) but also reduces total *dper* mRNA levels (Fig. 6b). Nonetheless, although changes in dmpi8 splicing efficiency have secondary consequences on *dper* mRNA levels^5,38^, at this stage we cannot rule out the possibility that B52 modulates the proportion of dmpi8-spliced transcripts by differential effects on the stabilities of the different *dper* isoforms (i.e., transcripts retaining the dmpi8 intron versus those where it was spliced).

Although we did not do an extensive analysis of different tissue-specific drivers, the results strongly suggest that the effects of B52 on mid-day sleep are mediated via its presence in clock cells (Figs 3 and 5). This is not surprising if the main effect of B52 on mid-day sleep is via directly modulating dmpi8 splicing since clock cells are the sites where *dper* (and *tim*) is expressed. There are approximately 150 ‘clock’ (or perhaps more precisely, *dper*/*tim*-expressing) neurons in the adult brain that are organized into several clusters that are spatially and functionally distinct^1,26,31^. Down-regulating *B52* in the small set of clock neurons defined by PDF-expression resulted in elevated mid-day sleep (Fig. 5b). The PDF-expressing clock neurons are not only critical for maintaining circadian behaviour^32^, but also have roles in arousal and sleep^33–36,42^, suggesting these cells are a key site-of-action for dmpi8 splicing in regulating daytime sleep. A recent study identified a dorsal set of clock neurons (DN1s) as having strong effects on mid-day siesta^43^. Since DN1s appear to be targets of PDF signalling^44^ it is possible that any effects of B52 on mid-day sleep levels via controlling dmpi8 splicing in PDF-expressing cells involves participation of complex neuronal circuitry. Future studies using *RNAi-B52* targeted to different subsets of clock and/or other sleep and arousal centers will be of interest.

Studies in *Drosophila* and mammals have shown that SR proteins can have partially redundant roles but also affect specific sets of splicing junctions (e.g.^19,21^). While we cannot rule out the possibility that SR proteins besides B52 can regulate dmpi8 splicing under certain conditions, our findings strongly suggest that regulation of dmpi8 splicing or mid-day siesta is not a shared feature of all SR proteins (Figs 1 and 5). A role for B52 in regulating dmpi8 splicing efficiency is consistent with prior work showing that this SR protein binds the 3′ UTR of *dper* (^19^; and see Fig. 2). B52 is an essential gene that is required for development and is the major SR protein expressed in the brain and eye (^25,45,46^; and data annotated from flybase.org). In addition, hundreds of splicing events are modulated by B52, indicating that this SR protein has many different targets^19^. At present, it is not clear if there are any functional relationships between the regulation of dmpi8 splicing efficiency by B52 and other pre-mRNA targets of B52.

As mentioned above, a transcriptome wide analysis of SR protein binding to transcripts expressed in S2 cells identified a B52 cross-linking site in the *dper* 3′ UTR that is situated 8 nt downstream of SNP4 (Fig. 1A)^19^. The B52 cross-linking site identified in the *dper* 3′ UTR is GAACC^19^ (Fig. 1A; and T. Bradley and M. Blanchette, personal communication). While it is not definitive that the B52 cross-linking site in the *dper* 3′ UTR is part of the actual B52 recognition motif or simply nearby, it is interesting that the region includes GAA. GAA repeat sequences have been shown to function as purine-rich exonic splicing enhances (ESEs) that stimulate splicing of introns with a weak 5′ss, 3′ss or both^47,48^, with a preference for acting downstream of weak 3′ss to stimulate splicing of the upstream intron^49–51^. Thus, it is possible that binding of B52 downstream of the dmpi8 intron enhances 3′ss recognition.

An interesting implication of our work is that B52 might have a role in the natural variation of mid-day sleep in *Drosophila*. The VT1.1 and VT1.2 haplotypes are commonly found in natural populations of *D. melanogaster* from the eastern coast of the United States^14^. Flies carrying the VT1.1 haplotype exhibit higher dmpi8 splicing efficiency and reduced mid-day siesta compared to those with the VT1.2 version^14^ (and Fig. 1). Using a cell culture system, we show that down regulation of *B52* has a generally stronger effect on reducing dmpi8 splicing in the VT1.1 haplotype compared to that of VT1.2 (Fig. 1). In agreement with this finding, B52 has increased binding to transcripts containing the VT1.1 haplotype of the *dper* 3′ UTR compared to that of VT1.2 (Fig. 2). Although these results were obtained using *Drosophila* S2 cells and further studies in flies will be required to better explore physiological significance, they suggest SNPs in the *dper* 3′ UTR modulate B52 binding.

How might the different SNP variants found in VT1.1 and VT1.2 affect B52 binding? Prior work showed that much of the difference in dmpi8 splicing and mid-day sleep levels observed between VT1.1 and VT1.2 containing flies are due to two closely spaced SNPs, SNP3 and SNP4^14^ (Fig. 1A). SNPs 3 and 4 are separated by 11 nt and we previously showed that the combination in VT1.1 (i.e., SNP3G and SNP3A) leads to higher dmpi8 splicing efficiency and reduced mid-day siesta compared to the VT1.2 version (i.e., SNP3A and SNP3G)^14^. Intriguingly, the VT1.1 version of SNP3 yields a GAA element as does the SNP4 variant (SNP3G, **G**AA; SNP4A, G**A**A; where the SNP variant is in bold and underlined). This is contrast to the VT1.2 haplotype (SNP3A, **A**AA; SNP3G, G**G**A). It is possible that the presence of additional GAA repeats^47,52^ around the B52 binding site in the VT1.1 haplotype is the reason for its enhanced binding to B52 (Fig. 2) and preferential stimulation of dmpi8 splicing efficiency by B52 (Fig. 1B).

Another possibility for the differential effects of B52 on dimpi8 splicing for transcripts containing either the VT1.1 and VT1.2 haplotypes is based on earlier work suggesting the B52 binding motif involves stem-loop structures^53^, although the physiological significance of this structure is not clear. Of potential interest, using several publicly available RNA folding prediction programs, the RNA sequences including SNPs 3, 4 and the B52 cross-linking site (GAACC) are predicted to form stem-loop structures that differ between VT1.1 and VT1.2 (data not shown). Clearly, future studies using fly models that examine the effects of B52 in combination with individual SNPs and potential GAA based ESEs in the *dper* 3′ UTR will be required to better evaluate these aforementioned possibilities.

Although the temperature dependency of dmpi8 splicing is based on suboptimal 5′ and 3′ss, a recent study showed that the small fluctuations in skin temperature observed in many mammals can drive rhythms in alternative splicing events via daily changes in SR protein phosphorylation^54^. Changes in the phosphorylated state of SR proteins can influence their sub-cellular localization and function^16,18,55^. Therefore, SR proteins can function in thermal sensing pathways, leading to temperature-dependent changes in alternative splicing pathways. It will be of interest to determine if a similar mechanism also operates in *Drosophila*. In summary, our findings identify a novel role for B52 in the regulation of mid-day sleep in *Drosophila* and suggest that B52 contributes to natural variation in sleep behavior via differential binding to the *dper* 3′ UTR as a consequence of SNP variations.

## Methods

### Fly strains and measuring daily wake-sleep behavior

All flies were routinely reared at room temperature (22–25 °C) and maintained in vials or bottles containing standard agar-cornmeal-sugar-yeast-Tegosept-media. All flies carrying the UAS-RNAi and Gal4 drivers used in this study were in the *w*^1118^ genetic background. The RNAi strains used in this study were obtained either from the Vienna Drosophila Resource Center (VDRC; *RNAi-B52*, V38860, V38862, V101740; *RNAi-Rbp1-like*, V51615; XL6, V31203) as originally described^27^, or the Bloomington Drosophila Stock Center (BDSC; *RNAi-B52*, T37519). They were crossed to flies with various tissue-specific drivers at 25 °C. These drivers were *tim(UAS)-Gal4* (TUG) as originally described^30^, *PDF-Gal4* (BDSC, stock #25031) and *Gmr-Gal4* (BDSC, stock #1104). For the Gal4 driver strains we first introduced the *UAS-dicer2* transgene by crossing schemes that involved transgenic flies carrying the UAS-dicer2 transgene (BDSC, stocks #24651 or #24650), which should enhance RNAi-mediated inhibition. The males of RNAi strains were crossed with virgins of the desired driver strain, and in a complementary cross, virgins of the different RNAi strains were crossed with males of the desired driver strains. In general, behavioral results were averaged for both reciprocal crosses (e.g., Table 1). In addition, we set up contemporaneous control crosses whereby each parental strain was crossed to *w*^1118^. Young adult male or female progeny (2–5 days old) were used for behavioral assays.

Daily wake-sleep behavior was continuously monitored and recorded using the Trikinetics system (Waltham, MA, USA) system, as previously reported^7,28^. Briefly, individual adult flies (2–5 day-old) were placed in 65 mm × 5 mm glass tubes containing 5% sucrose with 2% Bacto agar. Throughout the testing period flies were maintained at the indicated temperature (18° or 25 °C) and subjected to at least 5 days of 12 hr light: 12 hr dark cycles [LD; where zeitgeber time 0 (ZT0) is defined as lights-on]. Cool white fluorescent light (~1000 lux) was used during LD and the temperature did not vary by more than 0.5 °C between the light and dark periods. In general, after five days in LD, flies were kept in constant darkness (DD) for seven days. Data analysis of either locomotor activity or sleep parameters was done with the FaasX and Matlab programs, as previously described^7,11,28^. Sleep was defined as no detection of locomotor activity movement for any period of 5 contiguous min, which is routinely used in the field (e.g.,^29^). For each genotype and condition, activity data was pooled from multiple individual flies, and group averages are shown. In addition, for sleep values during LD, the data are an average of the last three LD days; for DD, the values were from single days. Free-running periods of locomotor activity rhythms were based on the data collected during six consecutive days in DD and using the FaasX program (kindly provide by F. Rouyer, France), as previously described^7^. P-values of significance were calculated by using two-tailed *t*-test or ANOVA, as indicated in the figure legends.

### Tissue culture constructs and dmpi8 splicing assay

The pAct-Luc-VT1.1 and pAct-Luc-VT1.2 plasmids were previously described^14^. To generate pMT-FLAG-B52 plasmid, coding regions of *B52* were amplified by PCR from a cDNA clone obtained from the Drosophila Genomics Resource Center (DGRC; the *B52*-cotaining plasmid used was GH20537). In addition, we used PCR to introduce a 3xFLAG followed by a 6xHis stretch upstream of the B52 open reading frame and subcloned the fragment into the pMT/V5-His vector (Invitrogen, ThermoFisher) using NotI and ApaI. All final constructs used in this study were validated by DNA sequencing prior to their further use.

Measurement of the dmpi8 splicing efficiency in *Drosophila* Schneider 2 (S2) cells was performed essentially as previously described^7^. Briefly, the S2 cells and Drosophila Expression System (DES) media were purchased from Invitrogen. S2 cells were transiently transfected using Effectene reagent (Qiagen), according to manufacturer’s instructions. Approximately 1.5 × 10^6^ S2 cells were placed in 6-well plates and transfected with 125 ng of either pAct-Luc-VT1.1 or pAct-Luc-VT1.2. After transfection, cells were allowed to recover for 2 days. Subsequently, cells were transferred to the indicated temperature for overnight incubation before harvesting. Cells were collected by centrifugation and washed twice with ice-cold PBS on ice. Total RNA was extracted using the TRI Reagent (SIGMA) according to manufacturer’s instructions. For each sample, about 1 µg of RNA was subjected to reverse transcription using oligo(dT), and dmpi8 splicing efficiency measured by semi-quantitative PCR in the presence of the forward primer P6869 (5′ TAGTAGCCACACCCGCAGT 3′) and the reverse primer P7197 (5′ TCTACATTATCCTCGGCTTGC 3′), as previously described^7,38^. The non-cycling Cap Binding Protein 20 (CBP20) gene was used as an internal control^7,38^, and was measured using the primers CBP294f (5′ TGA TTG TGA TGG GCC TGG ACA AGT 3′), and CBP536r (5′ GTC CAA GCG AGT GCC ATT CAC AAA 3′), as previously described^13^. PCR products were separated and visualized by electrophoresis on 2% agarose gels, and the bands were quantified using a Typhoon 9400 Imager. The values of *dper*-containing amplified products were normalized relative to CBP20 levels.

### dsRNA production and RNAi treatment is S2 cells

RNA interference (RNAi) in S2 cells was carried out using double-stranded RNA (dsRNA) as described previously^56^. Individual gene sequences were amplified by PCR from cDNA clones obtained from the Drosophila Genomics Resource Center (DGRC; the plasmids were, GH12433 for *B52*, GM02602 for *Rbp1*, LD40489 for *Sf2*, LD46359 for *Xl6*, RE39606 for *Rsf1* and LD32469 for *SC35*). The primers used in the PCR reactions contained a 5′ T7 RNA polymerase-binding site (5′-TTAATACGACTCACTATAGGGAGA-3′; T7 site is underlined) flanked by a gene-specific sequence. The sense and antisense gene-specific sequences are shown in Table S1. The PCR products were purified by using the QIAquick PCR Purification Kit (Qiagen) and used as templates to produce dsRNA with the MEGAscript T7 Transcription Kit (Ambion). The RNA products were treated with DNase I (Ambion) to digest template DNA, and extracted with MEGAclear^TM^ kit (Ambion). To ensure that most of the RNA products were in a double-stranded form, the RNA solution was heated at 65 °C for 30 min and then slowly cooled to room temperature. The quality and concentration of dsRNAs were checked by 1% agarose gel electrophoresis. DsRNAs were stored at −20 °C before use. RNA-mediated interference was applied to S2 cells as described previously^56^. After the addition of dsRNA, cells were left to recover for 2 days, transfected with either the pAct-Luc-VT1.1 or pAct-Luc-VT1.2 plasmids, and incubated for a further 1.5 days before cells were harvested.

### B52 binding studies

To measure binding of *Luc-dper* transcripts to B52, we followed a protocol that was generously provided by T. Bradley and M. Blanchette, based on their published work^19^. Essentially, S2 cells were maintained at 22 °C in DES media (Invitrogen) in 65 cm dishes, and each sample was done in triplicate. The cells were transiently transfected with 0.8 μg of different *dper* 3′UTR containing plasmids (pAct-Luc-VT1.1 and pAct-Luc-VT1.2) and 0.2 μg of pMT-FLAG-B52 or empty control pMT/V5-His plasmid were used. Expression of recombinant *B52* was induced by adding 500 μM CuSO_4_ to the culture media 36 h after transfection. Cells were gently washed twice with ice-cold PBS, and covered by 1.5 ml of PBS. The cells were placed on ice and the cover of each dish was removed before the cells were treated in a Stratagene stratalinker with 200mJ/cm^2^ of UV irradiation for 10 min. In addition, we also did a mock-treatment whereby cells were treated identically but were not exposed to UV irradiation. Cells were subsequently scraped, washed twice in cold PBS. The cell pellet was lysed with 350 μL IP Lysis Buffer (Pierce) containing complete EDTA-free protease inhibitor mixture (Roche Applied Science), DTT (1 mM final) and RNAseOUT (40 u/ml; Thermo Fisher). Lysates were incubated on ice for 5 min with periodic mixing, followed by centrifugation. The supernatant was divided into two fractions (100 μl for analysis of input material and 250 μl for immunoprecipitation). For immunoprecipitation of FLAG-tagged B52, 30 μl of anti-FLAG M2 magnetic beads (Sigma-Aldrich) were added to the lysate and incubated for 4 hr at 4 °C with constant shaking. The beads were washed three times with IP Lysis Buffer, resuspended in 250 μl of IP Lysis Buffer, and 10 μl removed to be analyzed by western blotting for B52. Subsequently, 100 μl of proteinase K mix [10 mM Tris, 1% SDS, 0.25 mM CaCl_2_, and 0.5 mg/ml Proteinase K (NEB)] was added and incubated at 55 °C for 15 min with constant gentle mixing. Beads were collected and bound RNA was purified by the RNeasy Micro Kit following the manufacturer’s instructions (Qiagen). The relative levels of input or B52-immunoprecipitated *Luc-dper* transcripts were quantified by qRT-PCR using the SuperScript First-Strand Synthesis System (ThermoFisher Scientific) in the presence of *luciferase*-specific primers, as follows; sense primer, 5′-AGCGACCAACGCCTTGATT-3′, and antisense primer, 5′-ACTTCAGGCGGTCAACGATG-3′.

To detect recombinant FLAG-B52 protein levels, 10 μl of either the input cell extract or the sample following immunoprecipitation were analyzed by immunoblotting, essentially as previously described^57^. Briefly, extracts were resolved by 10% SDS-polyacrylamide gel electrophoresis, transferred to nitrocellulose paper, and immunoblots probed with mouse anti-FLAG M2 monoclonal antibody (1:5000; Sigma). Immunoblots were visualized using ECL plus reagent (GE LifeSciences).

### Splicing assay in flies

The splicing efficiency of dmpi8 in flies was measured as previously described^7,14,38^, with some minor modifications. Briefly, vials containing ~40 young (2 to 5 day-old) adult flies were placed in controlled environmental chambers (Percival, USA) at the indicated temperature and exposed to five 12hr light: 12 hr dark cycles. Every four hours at selected times during the last day in LD, flies were collected by freezing and heads isolated. Total RNA was extracted and the relative splicing efficiency of dmpi8 measured using a semi-quantitative RT-PCR assay as previously described^7,14,38^. Briefly, RNA was collected from isolated fly heads using TRI-Reagent (SIGMA). Approximately 1 μg of total RNA was reverse transcribed using oligo(dT) and Thermoscript reverse transcriptase enzyme (Invitrogen or Clontech) in a 20 μl reaction. Gene specific primers flanking the 3′ UTR intron of *dper* were used to amplify both the spliced and unspliced forms in a 25 μl reaction using 1 μl of RT product as template. The following primers were used to amplify the target regions: sense primer P6851f (5′ ACA CAG CAC GGG GAT GGG TAG T 3′) and antisense primer P7184r (5′ GGC TTG AGA TCT ACA TTA TCC TC 3′)^13^. The non-cycling Cap Binding Protein 20 (CBP20) gene was used as an internal control. For CBP20, the sense primer is CBP294f (5′ TGA TTG TGA TGG GCC TGG ACA AGT 3′), and the antisense primer is CBP536r (5′ GTC CAA GCG AGT GCC ATT CAC AAA 3′)^13^. PCR products were separated and visualized by electrophoresis on 2% agarose gels containing Gelstar (Cambrex Co., USA), and the bands quantified using a Typhoon 9400 Imager. The values of *dper*-containing amplified products were normalized relative to CBP20 and expressed as the proportion with the 3′-terminal intron removed. We routinely collected samples after different PCR cycle lengths to ensure that the amplified products were in the linear range for quantification.

### Availability of materials and data

The datasets analyzed during the current study are available from the corresponding author on reasonable request. All the flies used in this study were previously reported and are available from public resources. All the plasmids used in this study are available on request from the corresponding author.

## Electronic supplementary material

Supplementary file
