# Supplementary material for: The SR protein B52/SRp55 regulates splicing of the period thermosensitive intron and mid-day siesta in Drosophila
Source: Sci Rep. 2018 Jan 30;8:1872. doi: 10.1038/s41598-017-18167-3 (PMC5789894; doi:10.1038/s41598-017-18167-3)
Supplement: Supplementary file 1 — Supplementary file [file 41598_2017_18167_MOESM1_ESM.pdf]

<u>Supplementary file (Table S1 and Figures S1 – S5)</u>

Title: The SR protein B52/SRp55 regulates splicing of the *period* thermosensitive intron and mid-day siesta in *Drosophila*

Authors: Zhichao Zhang, Weihuan Cao, and Isaac Edery

Table S1. Sense and antisense gene-specific sequences used to generate double-stranded DNA for RNAi studies in S2 cells

SR protein	cDNA clone ID (DGRC)[a]	Gene-specific sequence (5' - 3')[b]
B52	GH12433	F: AGTCCAAGTCGCCAGTCAAG
		R: CAATGTCCCATGTCCACAAC
Rbp1	GM02602	F: CCCGCAATACTCACGATACC
		R: ACTTCTGTGAGGCTGTGACG
SF2	LD40489	F: ATCGTGGCAAGATCTCAAGG
		R: GTTGGCTTCGGTGAGAGTTC
XL6	LD46359	F: TAAAATGAGTTTTGCGCTGC
		R: CGCACTCGTAGCACTTATCG
Rsf1	RE39606	F: GTATGGCAAGCTGAATTCGGTGTGG
		R: GTGGGGGAATGAGGGAATCTGTTGG
SC35	LD32469	F: AGCTCCGCGTACAGATGG
		R: AAGCCTATGTGCAAGAACCG

[a]DGRC, Drosophila Genomics Resource Center.
F, forward primer; R, reverse primer.

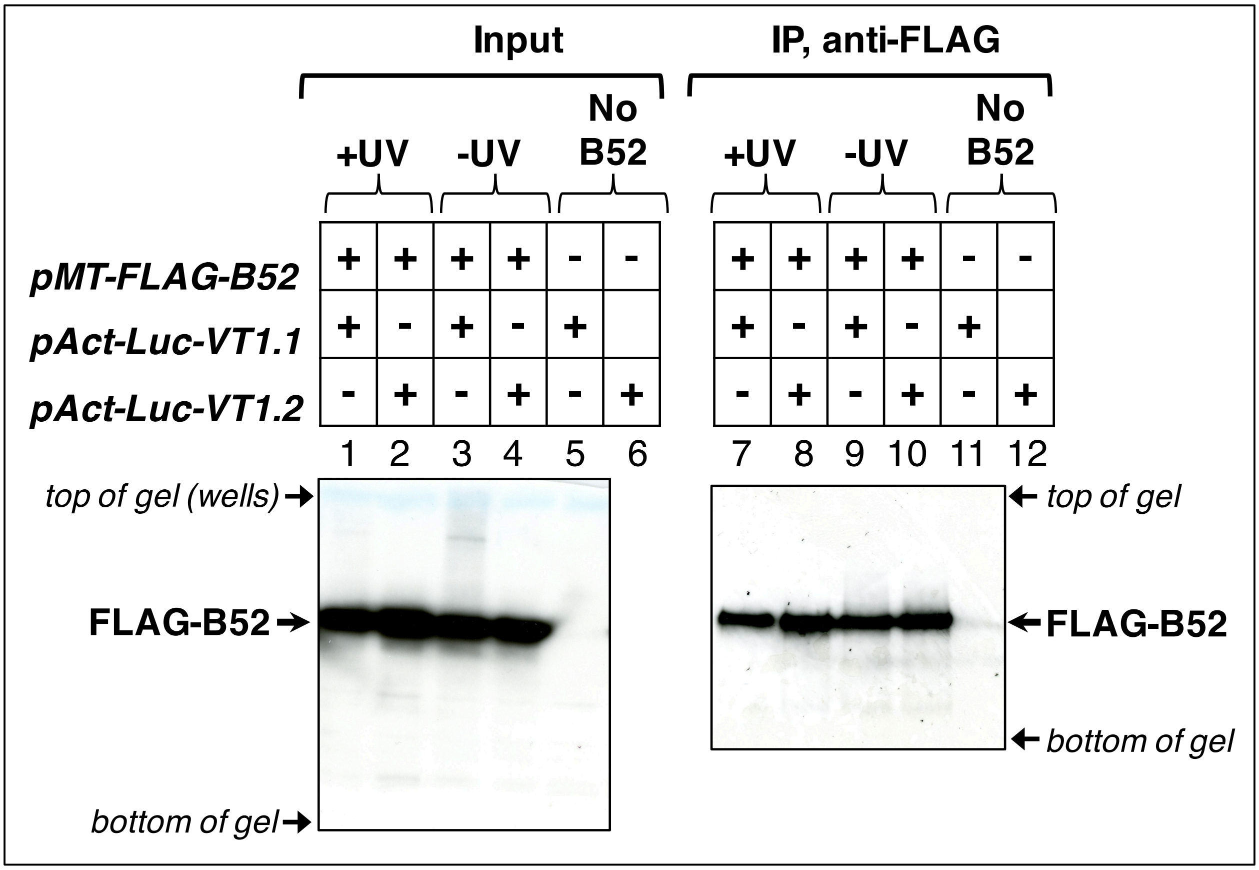

Figure S1. Similar amounts of FLAG-B52 were produced and immunoprecipitated in cells transfected with either Luc-VT1.1 or Luc-VT1.2 plasmids (related to figure 2)

Drosophila S2 cells were transfected with either the pAct-Luc-VT1.1 (VT1.1) or pAct-Luc-VT1.2 (VT1.2) plasmid, in the presence (+) or absence (-) of a plasmid expressing FLAG-B52, as indicated. After UV irradiation (+ UV) or mock-treatment (- UV), cells were homogenized in 350 μl of lysis buffer. A fraction of the cell extract (100 μl) was used to detect FLAG-B52 using immunoblotting (Input, lanes 1-5). The remainder of the cell extract was subjected to immunoprecipitation (IP) in the presence of anti-FLAG antibodies. Following IP, an aliquot was used for immunoblotting of B52-Flag (IP; lanes 7-11). For the immunoblots shown, the left panel (lanes 1-5) and the right panel (lanes 7-11) come from two different gels. Note that in the blots shown, FLAG-B52 was not analyzed by blotting for samples in lanes 6 and 12. Cropped images were used in figure 2.

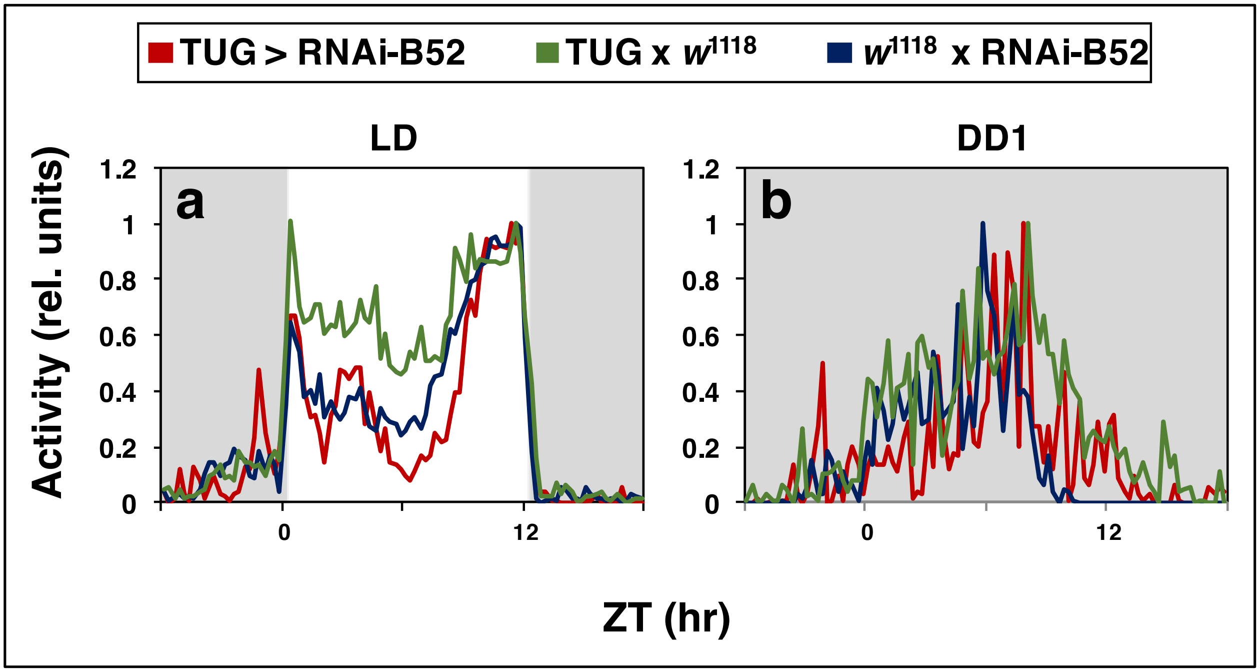

Figure S2. Daily activity profiles of flies with knock-down of B52 and control crosses (related to figures 3a and 4a)
Young male progeny from the indicated cross (top of panels) were kept at 18°C and entrained for 5 days in 12 hr:12 hr light/dark (LD) cycles [where Zeitgeber time (ZT) 0 is lights-on] followed by several days in constant darkness (DD). Shown are the daily locomotor activity profiles during LD (a) and during the first day of DD (b). The RNAi-B52 line used in this example is V101740, and the data are the same that were used to measure sleep levels in figure 3a and 4a. Activity levels are given in relative units. White shading, lights-on; gray shading, lights-off.

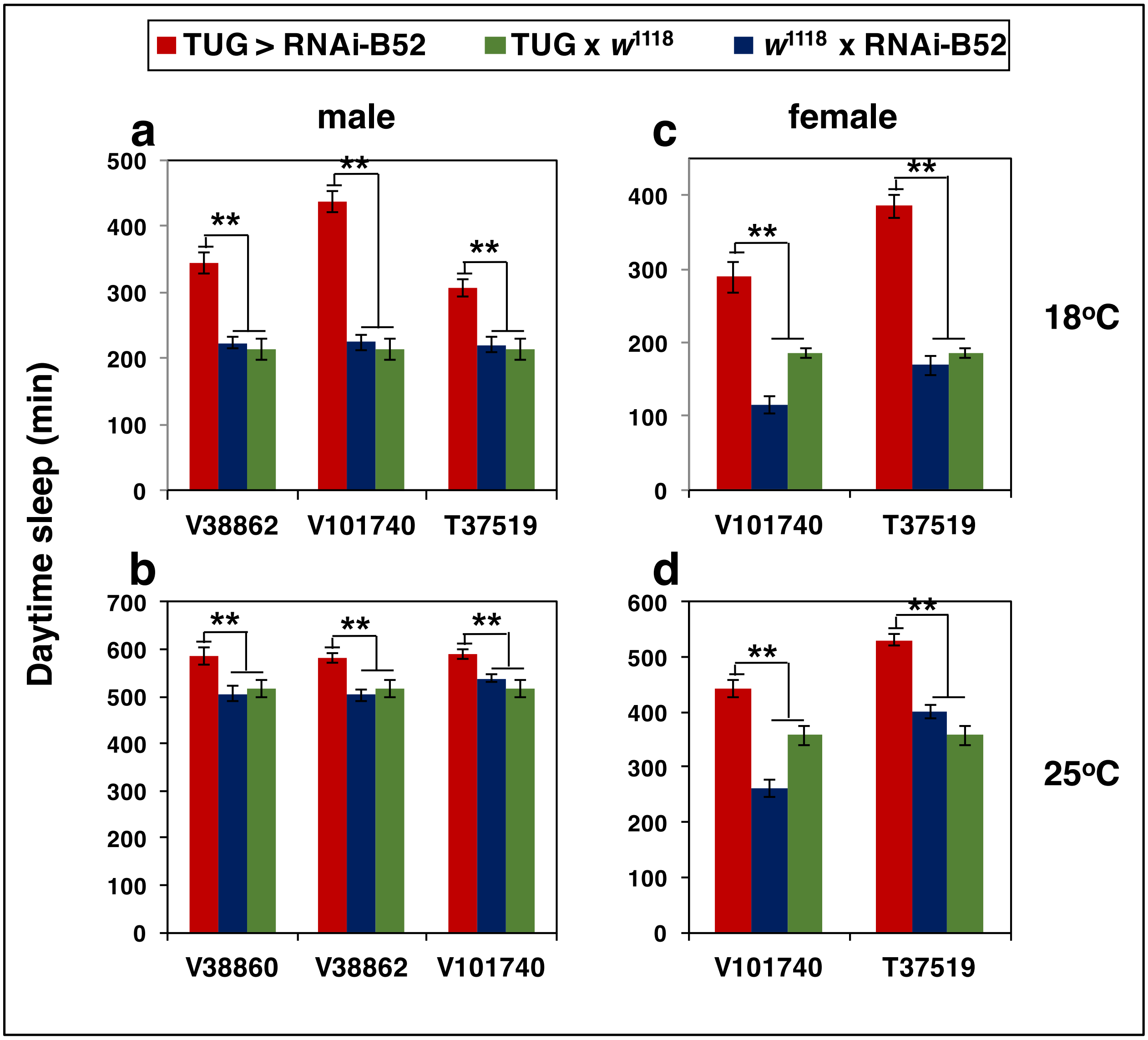

Figure S3. Knock down of *B52* in clock cells reduces daytime sleep in both sexes and several temperatures (related to figure 3)

(a-d) Young male (a, b) and female (c, d) progeny from the indicated cross (bottom of panels) were kept at 18°C (a, c) or 25°C (b, d) and entrained for 5 days in 12 hr:12 hr light/dark (LD) cycles [where Zeitgeber time (ZT) 0 is lights-on] followed by several days in constant darkness. Total daytime sleep levels were measured, and average values during the last 3 days of LD are shown. For each genotype, data from 16 individual flies was used. The data in panel a are the same as that shown in figure 3b. Although the majority of our studies were done with males at 18°C, knock down of *B52* significantly reduces daytime in males at 25°C and in females at both temperatures. Note that as previously reported males have higher mid-day sleep levels compared to females. Thus, at higher temperatures the already elevated mid-day sleep levels in males likely yields a 'ceiling' effect limiting the ability to observe further increases by silencing *B52*. Values for TUG>RNAi-B52 were significantly different compared to both control crosses; **, $p < 0.01$; two-tailed *t*-test. The following *p* values were determined: [Panel a, two-tailed *t*-test; TUG>V38862 vs V38862 x w^{1118}, 5.0 x 10^{-10}; TUG>V38862 vs TUG x w^{1118}, 1.2 x 10^{-8};

TUG>V101740 vs V101740 x w^{1118}, 1.68 x 10^{-20}; TUG>V101740 vs TUG x w^{1118}, 1.96 x 10^{-18}; TUG>T37519 vs T37519 x w^{1118}, 2.19 x 10^{-6}; TUG>T37519 vs TUG x w^{1118}, 1.06 x 10^{-8}]. [Panel b, two-tailed *t*-test; TUG>V38860 vs V38860 x w^{1118}, 7.9 x 10^{-4}; TUG>V38860 vs TUG x w^{1118}, 9.7 x 10^{-3}; TUG>V38862 vs V38862 x w^{1118}, 7.7 x 10^{-6}; TUG>V38862 vs TUG x w^{1118}, 7.0 x 10^{-3}; TUG>V101740 vs V101740 x w^{1118}, 2.8 x 10^{-5}; TUG>V101740 vs TUG x w^{1118}, 7.1 x 10^{-4}]. [Panel c, two-tailed *t*-test; TUG>V101740 vs V101740 x w^{1118}, 1.0 x 10^{-11}; TUG>101740 vs TUG x w^{1118}, 2.2 x 10^{-6}; TUG>T37519 vs T37519 x w^{1118}, 1.6 x 10^{-17}; TUG>T37519 vs TUG x w^{1118}, 3.9 x 10^{-20}]. [Panel d, two-tailed *t*-test; TUG>V101740 vs V101740 x w^{1118}, 1.4 x 10^{-12}; TUG>101740 vs TUG x w^{1118}, 3.5 x 10^{-4}; TUG>T37519 vs T37519 x w^{1118}, 6.8 x 10^{-13}; TUG>T37519 vs TUG x w^{1118}, 3.3 x 10^{-13}].

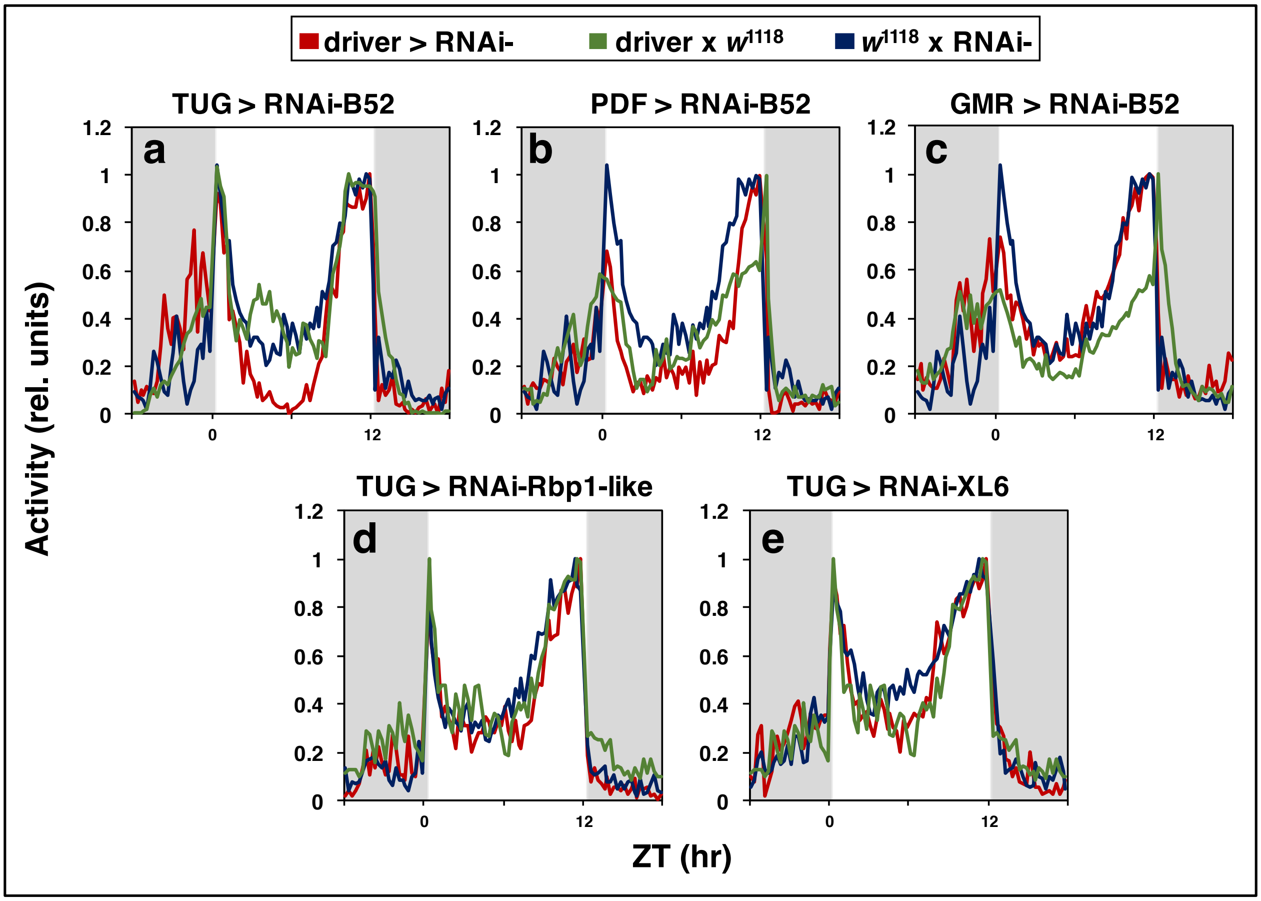

Figure S4. Daily activity profiles for sleep data shown in figure 5.
(a-e) Young male progeny from the indicated crosses (top of panels) were kept at 18°C and entrained for 5 days in 12 hr:12 hr light/dark (LD) cycles followed by several days in constant darkness (DD). Shown are the daily locomotor activity profiles (averaged over the last 3 days of LD). For each genotype, data from 16 individual flies was used to generate the graphs shown in each panel. The driver and SR protein targeted by RNAi are indicated. For RNAi-B52, the V101740 line is shown. The data are the same that were used to measure sleep levels in figure 5. Activity levels are given in relative units. White shading, lights-on; gray shading, lights-off.

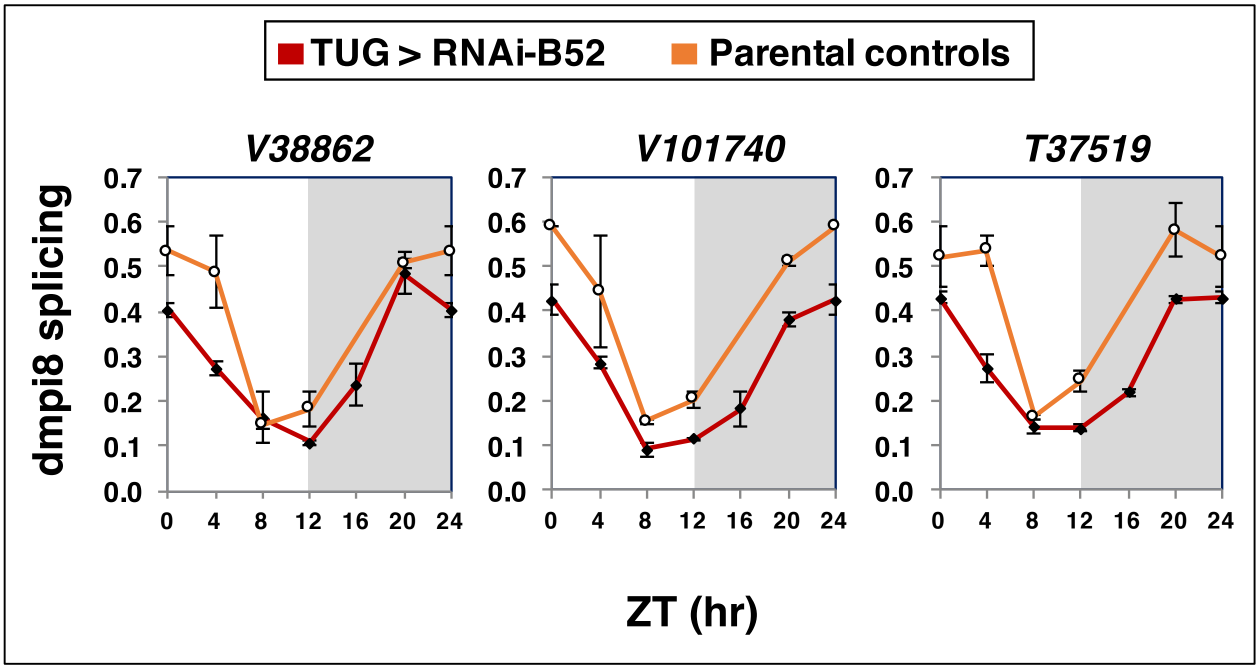

Figure S5. B52 stimulates dmpi8 splicing in flies (related to figure 6)
Young adult flies from the indicated cross and both parental controls (top of panels) were placed in 6 vials, and entrained for 5 days in 12 hr:12 hr light/dark (LD) cycles at 25°C. On the last day of LD, at the indicated times, one vial from each genotype was collected by freezing. Total RNA was extracted from fly heads and the splicing efficiency of dmpi8 calculated. For each *RNAi-B52* parental control, the values at each time-point were averaged with the TUG parent as they were similar (data not shown), leading to a separate parental average for each *RNAi-B52* line. Values shown are from the average of two independent experiments. Note the values for ZT0 were re-plotted for ZT24. Because the results were highly similar for the different RNAi-B52 lines, the results were pooled and shown in figure 6.
